# Estimating Frequency of Probable Autochthonous Cases of Dengue, Japan

**DOI:** 10.3201/eid2409.170408

**Published:** 2018-09

**Authors:** Akiyoshi Senda, Anavaj Sakuntabhai, Shinako Inaida, Yoann Teissier, Fumihiko Matsuda, Richard E. Paul

**Affiliations:** Kyoto University Faculty of Medicine, Kyoto, Japan (A. Senda);; Kyoto University Graduate School of Medicine, Kyoto (S. Inaida, F. Matsuda);; Pasteur Kyoto International Joint Research Unit for Integrative Vaccinomics, Kyoto (A. Sakuntabhai, F. Matsuda, R.E. Paul);; Institut Pasteur, Paris, France (A. Sakuntabhai, R.E. Paul);; Centre National de la Recherche Scientifique, Paris (A. Sakuntabhai, R.E. Paul);; Institut Louis Malardé, Papeete, French Polynesia (Y. Teissier)

**Keywords:** dengue, autochthonous, surveillance, outbreak threshold, epidemic, vector-borne infections, viruses, Japan

## Abstract

Imported dengue into naive areas is a recognized but unquantified threat. Differentiating imported and autochthonous cases remains problematic. A threshold approach applied to Japan identified several aberrant incidences of dengue. Despite these alerts, no epidemics occurred other than 1 in Yoyogi Park in Tokyo, which was probably an unusual event.

Dengue is a major international public health concern, and the number of dengue outbreaks has escalated over the past decade (*1*). International travel will ensure importation of dengue virus (DENV) from dengue-endemic regions into nonendemic countries (*2*). The potential threat of DENV invasion into naive areas is illustrated by autochthonous dengue cases in France and the United States (*3*,*4*) and unprecedented epidemics in the Madeira Islands of Portugal and Tokyo, Japan (*5*,*6*). Most human DENV infections are asymptomatic (*7*), but the virus can still be transmitted to mosquitoes (*8*), so repeated “silent” DENV invasion will probably become increasingly frequent.

In 2012, the World Health Organization released a global strategy for dengue prevention and control, with the objective of reducing dengue-attributable deaths by 50% and dengue-attributable illness by 25% by 2020 (*9*). These reductions are to be achieved, at least in part, by implementing improved outbreak prediction and detection through coordinated epidemiologic and entomologic surveillance. This approach is also important for areas where dengue is nonenedemic that have no defined surveillance strategy.

Within this context, we examine the case of Japan, which had an unprecedented autochthonous DENV type 1 epidemic in Yoyogi Park in Tokyo in 2014 and is experiencing an ever-increasing number of dengue cases. Analyzing dengue case surveillance data from a 6-year period, we assess whether other incidents occurred when the number of dengue cases exceeded the expected number because of importation and whether these incidents represented potential foci of epidemics. We discuss whether the Tokyo epidemic was a rare event, the probability of a repeat epidemic, and the value of establishing a dengue alert threshold.

## The Study

Since 1999, dengue has been 1 of the notifiable diseases under national surveillance across Japan. The case definition of dengue fever includes the presence of suspicious clinical symptoms and laboratory confirmation. The definition of an imported case is DENV infection in a patient who had traveled to a dengue-infected area within 2 weeks before symptom onset; all other cases are defined as autochthonous. All diagnosed dengue cases are registered in the surveillance system database (*10*).

We selected as study sites the 2 largest metropolitan areas, Greater Tokyo (including the prefectures of Tokyo, Saitama, Kanagawa, and Chiba) and Greater Osaka (including the prefectures of Osaka, Hyogo, and Kyoto), which encompass the largest number of dengue cases during the study period and can be considered as work commuting zones. Incidence rate was the number of cases divided by the population according to the 2015 national census. To calculate the threshold, we extracted data from annual reports for 2005 through 2014 (http://survey.tokyo-eiken.go.jp/epidinfo/csvinfo.do). We used Tukey’s box plot method to establish the median background weekly incidence of dengue in each study area based on the previous 6 years’ data. We defined the weekly threshold as the rounded-up value of the third quartile + 1.5 times the interquartile range of the number of cases from the same week. We applied the 6 previous years’ weekly thresholds of cases in each prefecture to the weekly reported cases for 2011–2016. We defined an outlier as a week when the number of cases was >1 above or equal to the threshold. We defined the threshold for an autochthonous epidemic alert as >2 consecutive weeks in which outliers were detected.

**Figure 1:**
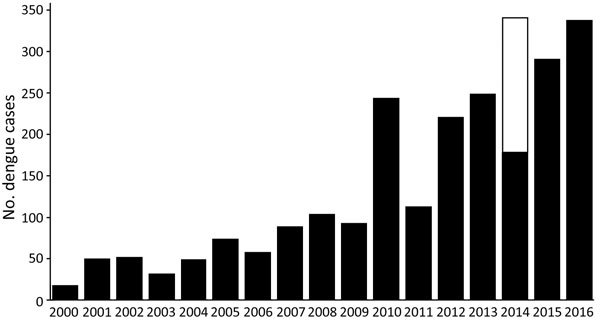
Annual reported dengue cases reported in dengue surveillance system, Greater Tokyo and Greater Osaka areas, Japan, 2011–2016. Black indicates imported cases; white indicates autochthonous cases detected during Tokyo epidemic.

The number of imported cases in Japan has been rising steadily over the past decade (Figure 1), concomitant with the increase in visitors, especially from South Korea, China, Taiwan, and Thailand (Technical Appendix Figures 1, 2). Until 2015, the number of outbound travelers from Japan exceeded that of inbound foreign travelers; one third of travelers from Japan went to dengue-endemic countries.

**Figure 2:**
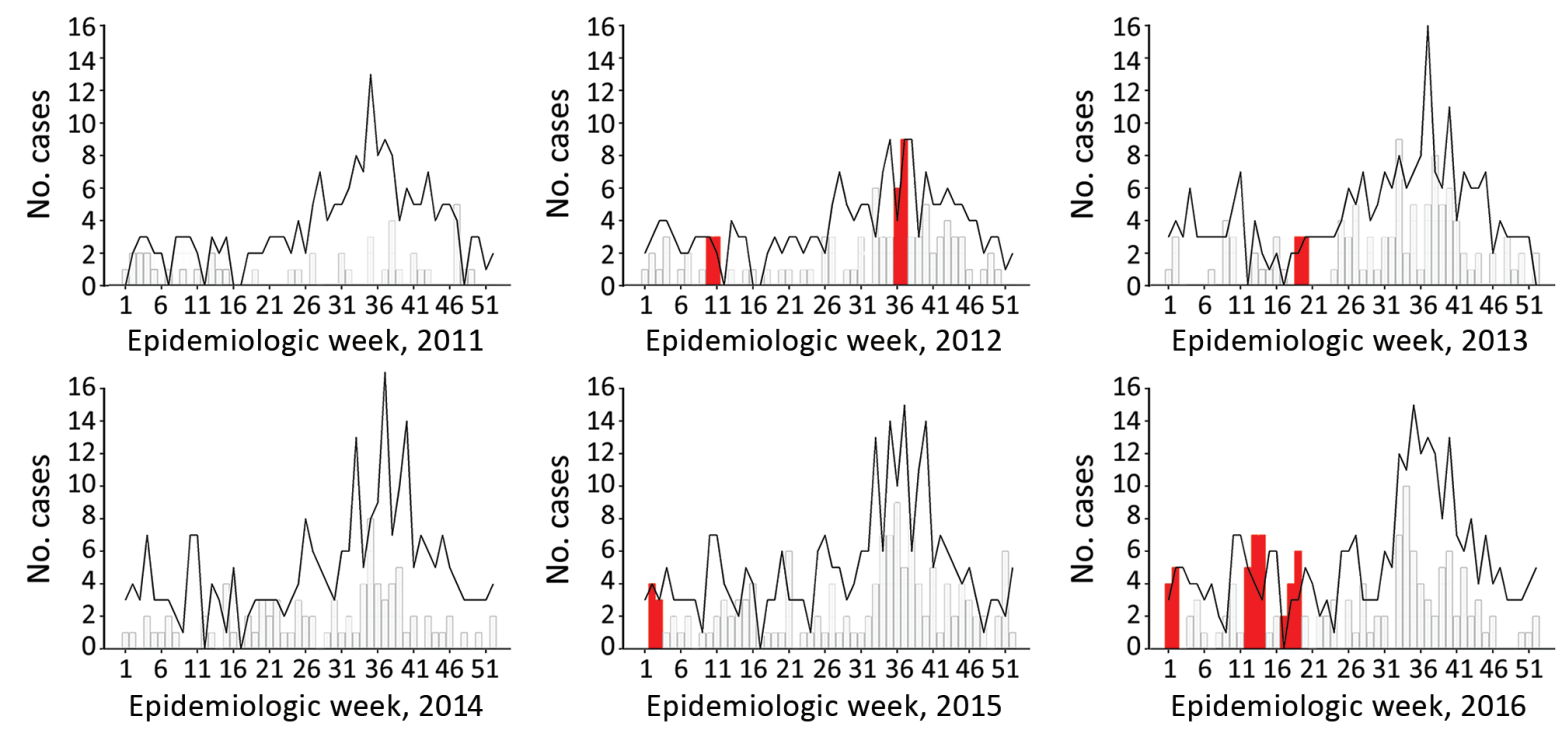
Detection of conditions warranting an autochthonous dengue case alert (red bars) compared with number of reported dengue cases per week (histogram) and estimated background threshold (black line), by year, Greater Tokyo area, Japan, 2011–2016.

In the Greater Tokyo and Greater Osaka areas, the threshold value of the incidence rate varied by year and place (Table). Outlying dengue case weeks were detected in all 7 prefectures of the 2 aggregated greater areas during the 6-year study period (Table; Figure 2). We noted several occasions when outliers were reported for 2 consecutive weeks (7 times in Greater Tokyo and 4 times in Greater Osaka). In Greater Tokyo, conditions warranting an alert occurred in 2012 (weeks 10–11 and 36–37), 2013 (weeks 19–20), 2015 (weeks 2–3), and 2016 (weeks 1–2, 12–14, and 17–19); in Greater Osaka, conditions warranting an alert occurred in 2012 (weeks 34–36), 2013 (weeks 41–42), 2014 (weeks 12–15), and 2016 (weeks 12–13).

**Table:** Thresholds and conditions warranting an autochthonous dengue case alert, Greater Tokyo and Greater Osaka areas, Japan, 2011–2016

Area and prefecture	Population	Total no. cases	Incidence rate, cases/10^6^ person-years	Threshold range, maximum (mean)	No. outliers	No. occurrences of alert conditions
Greater Tokyo area	36,126,355	609	2.8	17 (4.5)	52	7
Tokyo	13,513,734	358	4.4	11 (3.1)	32	2
Kanagawa	9,127,323	111	2.0	5 (0.77)	16	1
Saitama	7,261,271	45	1.0	3 (0.12)	4	0
Chiba	6,224,027	95	2.5	7 (0.89)	14	1
Greater Osaka area	16,986,037	230	2.3	9 (2.0)	25	4
Osaka	8,838,908	140	2.6	5 (1.4)	12	1
Hyogo	5,536,989	52	1.6	4 (0.29)	6	0
Kyoto	2,610,140	38	2.4	3 (0.24)	1	0

At the prefecture level, an alert condition was detected August 25–September 7, 2014, in Kanagawa Prefecture; this timing coincided with the Tokyo autochthonous epidemic. In addition, alert conditions were identified in Tokyo in 2013 (weeks 32–33) and 2016 (weeks 13–14), in Chiba in 2016 (weeks 33–34), and in Osaka in 2016 (weeks 12–13). The Tokyo and Osaka 2016 alert conditions occurred during the cold season and were probably caused by an increase in imported cases from Indonesia (*11*). By contrast, the alert condition in Chiba was followed by 2 additional cases reported in week 39 and 2 in week 42; both occurrences are outliers but are not in consecutive weeks and thus do not warrant an alert. The alert condition in 2013 in Tokyo was the first such occurrence observed in our data and coincided with a visit by a traveler from Germany who was allegedly infected with dengue in Japan (*12*) and had visited Tokyo.

## Conclusions

We have addressed the increasing probability of dengue invasion into Japan in light of the 2014 Yoyogi Park epidemic. Although the increase in dengue cases in Japan is concomitant with the increase in human travel between Japan and dengue-endemic areas, several reports exist of travelers contracting dengue while visiting Japan, which suggests that DENV is circulating in the form of subclinical infections; by extrapolation, allegedly imported cases might be autochthonous (*12*).

Differentiating imported and autochthonous cases based on recent travel history might be misleading. A case-patient in Hyogo Prefecture, ≈400 km from Tokyo, had stayed in Malaysia during the 12 days before symptom onset but had recollection of mosquito bites 6 days before onset, and the virus strain 100% matched the Yoyogi Park strain (*13*). Unusual above-threshold incidences of dengue might provide an additional criterion for differentiation. Although no official epidemic coincided with the occurrence of dengue in the traveler from Germany (*12*), our alert threshold pinpointed this period as being aberrant. Unusual above-threshold dengue incidences were noted during several periods, but no subsequent epidemic progression was noted. A substantial stochastic dieout of circulating DENV is occurring, despite permissive temperatures that would enable efficient transmission of DENV by the predominant mosquito vector, *Aedes albopictus*, which occurs at high densities in urban areas of Japan (*14*). However, most infections probably will go unnoticed, and the actual spread of DENV is greater than estimated from surveillance. DENV seroprevalence results for 207 persons who frequented Yoyogi Park indicated that 10 persons without recollection of symptoms were seropositive (*15*).

In conclusion, although increased human movement and permissive temperatures pose a threat for DENV invasion, evidence suggests that the Yoyogi Park epidemic was an exception and that a considerable viral biomass after repeated introduction might be required for successful viral implantation. The added utility of using a threshold approach to detect aberrant incidence rates for public health activities remains to be developed but could provide a basis for performing seroprevalence studies around cases detected during weeks with aberrantly high incidence to establish the extent of the problem.

Technical Appendix Overseas visitors to Japan, by year and by country of origin.

## References

[1] World Health Organization. Dengue and dengue hemorrhagic fever, fact sheet 117 [cited 2016 Oct 18]. http://www.who.int/mediacentre/factsheets/fs117/en

[2] Chen LH, Wilson ME. The role of the traveler in emerging infections and magnitude of travel. [xi.]. Med Clin North Am. 2008;92:1409–32, xi. 10.1016/j.mcna.2008.07.005 19061759 PMC7094659

[3] La Ruche G, Souarès Y, Armengaud A, Peloux-Petiot F, Delaunay P, Desprès P, et al. First two autochthonous dengue virus infections in metropolitan France, September 2010. Euro Surveill. 2010;15:19676. 20929659

[4] Murray KO, Rodriguez LF, Herrington E, Kharat V, Vasilakis N, Walker C, et al. Identification of dengue fever cases in Houston, Texas, with evidence of autochthonous transmission between 2003 and 2005. Vector Borne Zoonotic Dis. 2013;13:835–45. 10.1089/vbz.2013.1413 24107180 PMC3868290

[5] Alves MJ, Fernandes PL, Amaro F, Osório H, Luz T, Parreira P, et al. Clinical presentation and laboratory findings for the first autochthonous cases of dengue fever in Madeira island, Portugal, October 2012. Euro Surveill. 2013;18:20398. 23410256

[6] Kutsuna S, Kato Y, Moi ML, Kotaki A, Ota M, Shinohara K, et al. Autochthonous dengue fever, Tokyo, Japan, 2014. Emerg Infect Dis. 2015;21:517–20. 10.3201/eid2103.141662 25695200 PMC4344289

[7] Bhatt S, Gething PW, Brady OJ, Messina JP, Farlow AW, Moyes CL, et al. The global distribution and burden of dengue. Nature. 2013;496:504–7. 10.1038/nature12060 23563266 PMC3651993

[8] Duong V, Lambrechts L, Paul RE, Ly S, Lay RS, Long KC, et al. Asymptomatic humans transmit dengue virus to mosquitoes. Proc Natl Acad Sci U S A. 2015;112:14688–93. 10.1073/pnas.1508114112 26553981 PMC4664300

[9] World Health Organization. Global strategy for dengue prevention and control 2012–2020 [cited 2016 Oct 25]. http://www.who.int/immunization/sage/meetings/2013/april/5_Dengue_SAGE_Apr2013_Global_Strategy.pdf

[10] National Institute of Infectious Diseases. Annual report of Infectious Diseases Weekly Report [cited 2016 Oct 18]. http://www.nih.go.jp/niid/ja/survei/2270-idwr/nenpou/6141-kako2014.html

[11] National Institute of Infectious Diseases. Notification trends among imported dengue cases in Japan [cited 2016 Oct 18]. http://www.nih.go.jp/niid/ja/id/690-disease-based/ta/dengue/idsc/6663-dengue-imported.html

[12] Schmidt-Chanasit J, Emmerich P, Tappe D, Günther S, Schmidt S, Wolff D, et al. Autochthonous dengue virus infection in Japan imported into Germany, September 2013. Euro Surveill. 2014;19:20681. 10.2807/1560-7917.ES2014.19.3.20681 24480059

[13] Tajima S, Nakayama E, Kotaki A, Moi ML, Ikeda M, Yagasaki K, et al. Whole genome sequencing-based molecular epidemiologic analysis of autochthonous dengue virus type 1 strains circulating in Japan in 2014. Jpn J Infect Dis. 2017;70:45–9. 10.7883/yoken.JJID.2016.086 27169954

[14] Kobayashi M, Komagata O, Yonejima M, Maekawa Y, Hirabayashi K, Hayashi T, et al. Retrospective search for dengue vector mosquito *Aedes albopictus* in areas visited by a German traveler who contracted dengue in Japan. Int J Infect Dis. 2014;26:135–7. 10.1016/j.ijid.2014.06.005 25063022

[15] National Institute of Infectious Diseases. Report on the results of active epidemiological investigation of dengue autochthonous infection cases. Infectious Diseases Weekly Report [cited 2016 Dec 12]. http://www.nih.go.jp/niid/ja/id/693-disease-based/ta/dengue/idsc/iasr-news/5754-pr4252.html

