# Supplementary material for: Estimating Frequency of Probable Autochthonous Cases of Dengue, Japan
Source: Emerg Infect Dis. 2018 Sep;24(9):1705–8. doi: 10.3201/eid2409.170408 (PMC6106439; doi:10.3201/eid2409.170408)
Supplement: Technical Appendix — Overseas visitors to Japan, by year and by country of origin. [file 17-0408-Techapp-s1.pdf]

Article DOI: https://doi.org/10.3201/eid2409.170408

# Estimating Frequency of Probable Autochthonous Cases of Dengue, Japan

## Technical Appendix

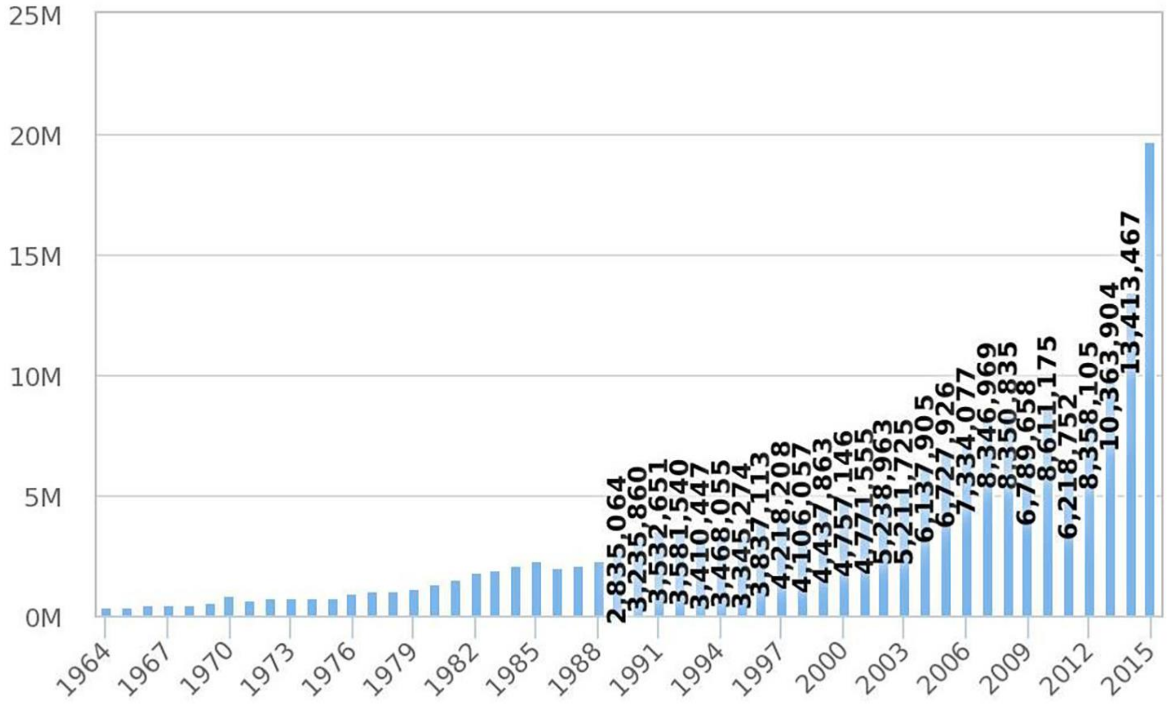


**Technical Appendix Figure 1.** Overseas visitors to Japan per year (http://www.tourism.jp/en/tourism-database/stats).

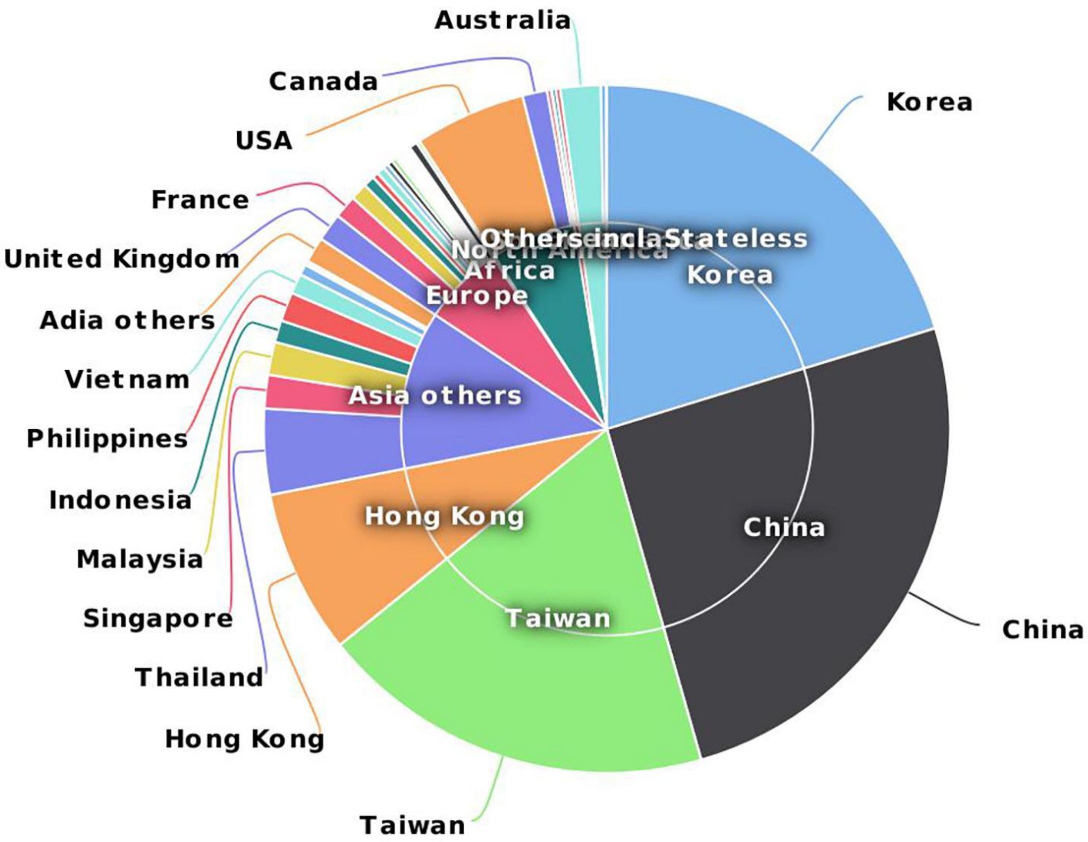


**Technical Appendix Figure 2.** Overseas visitors to Japan in 2015, by country of origin (http://www.tourism.jp/en/tourism-database/stats).
